# Diallyl disulphide inhibits apolipoprotein(a) expression in HepG2 cells through the MEK1-ERK1/2-ELK-1 pathway

**DOI:** 10.1186/s12944-017-0616-1

**Published:** 2017-11-25

**Authors:** Xiaofeng Ma, Yami Liu, Yanmei Tan, Kai Qu, Xinglan He, Hai Zhang, Zuo Wang

**Affiliations:** 1 Department of Cardiology, Affiliated Nanhua Hospital of University of South China, Hengyang, 421001 China; 2 Institute of Cardiovascular disease, Key Laboratory for Atherosclerology of Human Province, University of South China, Hengyang, 421001 China; 3 Department of Pathology, Changde Vocational Technical College, Changde, 415000 China; 4 Women and Children Healthcare Hospital of Zhu zhou, Zhuzhou, 412000 China; 5 Department of Pathology, The First Affiliated Hospital of University of South China, Hengyang, 421001 China

**Keywords:** Lipoprotein(a), Apolipoprotein(a), Diallyl disulfide, Extracellular regulated protein kinases, Mitogen-activated protein kinases, HepG2 cell

## Abstract

**Background:**

Lipoprotein(a) [LP(a)] is implicated as a common and independent risk factor for cardiovascular diseases. The therapeutic options currently available for reducing plasma LP(a) concentrations are limited. Diallyl disulphide (DADS), the main component of garlic, regulates lipid metabolism in hepatocytes and adipocytes through ERK1/2 signalling. This study aimed to assess the effect of DADS on apolipoprotein(a) [apo(a)] in HepG2 cells. We also determined the effects of DADS on apo(a) expression and secretion in HepG2 cells as well as the underlying mechanisms.

**Methods:**

We examined the role of DADS on apo(a) expression in HepG2 cells by treating cell with different concentrations of DADS (10, 20, 40 and 80 μg/mL) for 24 h or treating cells with 40 μg/mL DADS for 0, 6, 12, 24 and 48 h. Then we used quantitative real-time PCR to analysis apo(a) mRNA levels, used Western blot to analysis apo(a) protein levels and used enzyme-linked immunosorbent assay to test apo(a) secreted levels. To farther determined the role of DADS, we applied Transfection of small interfering RNA to knockdown ELK-1levels and applied PD98059, a specific inhibitor of ERK1/2, to block ERK1/2 signal.

**Results:**

The results show DADS inhibited apo(a) at both the mRNA and protein levels in HepG2 cells in a dose-dependent manner. DADS-mediated inhibition of apoa(a) expression in HepG2 cells was attenuated when the cells were cultured in medium containing PD98059 (ERK1/2 inhibitor) or were transfected with siRNAs against MEK1 or ELK-1. Overexpression of apo(a) yielded similar results.

**Conclusions:**

This study reveals that DADS can downregulate apo(a) expression in a dose-dependent manner via the MEK-ERK12-ELK-1 pathway.

## Background

Lipoprotein(a) [LP(a)] [1], which was first detected in 1963 by Kare Berg as a unique lipoprotein in humans, is a low-density lipoprotein (LDL)-like particle with a lipid core and apolipoprotein B (apoB). LP(a) contains a unique apolipoprotein, apo(a), which is tethered to apoB by a covalent disulfide bond [2, 3]. Although LP(a) was discovered over 50 years ago, the biological functions of this protein remain unclear. LP(a) can only be detected in humans, primates and hedgehogs [4], with levels ranging from <1 mg/dL to >100 mg/dL and are up to 90% genetically detected [5]. Epidemiological studies showed that plasma LP(a) concentrations >50 mg/dL, which is 80% of the maximum level seen in most populations, are positively associated with cardiovascular disease (CVD) risk [6, 7]. Increased LP(a) concentrations may increase the risk of CVD through prothrombotic or anti-fibrinolytic effects because apo(a) possesses structural homology to plasminogen and plasmin but exhibit no fibrinolytic activity. This risk is also alleviated through accelerated atherogenesis as a result of internal deposition of LP(a) cholesterol [8]. LP(a) levels are genetically determined and dependent mainly on the rate of hepatic synthesis or assembly of the apo(a) kringle moiety, which is non-covalently linked to LDL apoB on the hepatic cell surface [9].

Decreasing LP(a) levels are somewhat refractory to lifestyle or drug intervention; the use of nicotinic acid as specific therapeutic medication could decrease LP(a) concentrations by 15% to 25% [10, 11]. Apo(a), a specific component of LP(a), is exclusively synthesised in hepatic cells [12]. Apo(a) is highly homologous to plasminogen containing multiple copies of plasminogen kringle 4 and a single copy of plasminogen kringle 5, whose parent molecule is serine protease plasmin [13]. Apo(a) represents a heterogeneous class of glycoproteins, with molecular mass ranging from 280 kDa to 700 KDa [14]. Studies have indicated that the number of kringle 4 domains can range from 12 to 51 and together they can generate 34 differently sized apo(a) isoforms, whose weight varies between 200 and 800 KDa and are inversely correlated with LP(a) plasma levels [15]. Therefore, LP(a) concentrations can be controlled by regulating the rate of apo(a) de novo biosynthesis.

Diallyl disulphide (DADS, CH_2_ = CH–CH_2_–S–S–CH_2_CH = CH_2_), is an oil-soluble organosulphur compound isolated from *Allium sativum* [16]. The cardiovascular-protective effects of DADS have been extensively studied in recent years [17–19]. Garlic extracts can decrease cholesterol and plasma lipid levels in rats [20, 21], swine [22], rabbits [23] and chickens [24]. Intervention studies have shown that DADS significantly reduces the concentrations of plasma lipids, especially low-density lipoprotein cholesterol (LDL-C) and total cholesterol (TC), in humans [25]. However, several studies have showed that garlic supplementation does not decrease plasma cholesterol concentrations in humans [17, 26, 27]. *A. sativum* contains various amino acids, vitamins and organosulphur compounds, such as DADS, ajoene and S-allylycysteine, that may be responsible for the lipid-lowering properties of garlic. Previous studies showed that DADS exerts its effects via the ERK signalling pathway [28], which is a key pathway for regulating the metabolism of glucose and lipid in adipose tissues [29]. Moreover, the apo(a) promoter contains an Ets-1 binding motif that binds to ELK-1 and inhibits apo(a) transcription. ELK-1 is a well-characterised common nuclear substrate, belongs to the family of Ets domain-containing transcription factors and it is activated by ERK1/2. Thus far, the exact mechanism through which DADS reduces LP(a) concentration in plasma has remained unclear. In the study, we hypothesise that DADS suppresses LP(a) levels in HepG2 cells via the MEK1-ERK1/2-ELK1 signalling pathway.

## Methods

### Dads

DADS (Fluke) and Tween-80 were dissolved at a ratio of 1:2, and diluted 100-fold in saline before storing at −20 °C.

### Cell culture

Human hepatoma cell line HepG2 was purchased from the Chinese Academy of Shanghai Institute for Cell Biology. HepG2 cells were cultured in DMEM/high glucose (HyClone) supplemented with 10% fetal bovine serum (FBS) under standard culture conditions (37 °C, 95% humidified air and 5% CO_2_). The cells were cultured without serum for at least 6 h prior to the experiments.

### Transfection of small interfering RNA

Short-interfering RNA (siRNA) specific for ELK-1 was purchased from Santa Cruz Biotechnology. Control siRNA targeting the red fluorescent protein (CCACTACCTGAGCA-CCCAG) was used as negative control. siRNAs were transfected into HepG2 cells in six-well cell plates (2 × 10^6^ cells/well) diluted with Lipofectamine 2000 reagent. Twenty-four hours after transfection, the ELK-1siRNA suppressed the expression of ELK-1 proteins by 72% compared with the control siRNA, according to Western blot analysis.

### RNA isolation and quantitative real-time PCR analysis

Total RNA was extracted using TRIzol reagent (TIANGEN) according to the manufacturer’s instructions. RNA was reverse transcribed to cDNA by using a ReverAidTM First-strand cDNA synthesis kit. Real-time quantitative PCR (RT-qPCR) was performed on a Real-Time PCR System (version 3.5, Roche). Crossing point and melting curve for each reaction were analysed using the LightCycler software. GAPDH was used as the internal control. Data were analysed by 2-ΔΔCt.

### Detection with enzyme-linked immunosorbent assay (ELISA)

LP(a) ELISA kit was obtained from biohjsw (Xiamen, China). The levels of LP(a) secretion were determined by ELISA with a commercial reagent kit following the manufacturer’s instructions.

### Western blot analysis

Total proteins were extracted from isolated cells by using RIPA buffer and 1 mmol/L phenylmethyl sulfonyl fluoride (94:6). The proteins were separated by sodium dodecyl sulfate polyacrylamide gel electrophoresis and transferred onto a polyvinylidene difluoride membrane. The membrane was incubated with the primary antibodies against apo(a), MEK, ERK1/2, p-ERK1/2, ELK-1 and p-ELK-1 at room temperature for 4 h. After washing with Tris-buffered saline with Tween 20 three times, the membrane was incubated with secondary antibodies conjugated with horseradish peroxidase for 2 h. The proteins were visualised using chemiluminescence (ECL Plus Western Blot Detection System; Amerisham Biosciences, Foster City, CA, USA).

### CHIP assay

The ChIP assay was performed with HpeG2 cells treated with DADS for 24 h using EpiQuik Chromatin Immunoprecipitation (ChIP) Kit (Epigentek) according to the manufacturer’s instructions, with minor modifications. The nuclei were extracted and sonicated to yield 500 to 1000 bp DNA fragments. Aliquots of sheared chromatin were then immunoprecipitated using 4 μg anti-ELK1 or 1 μg anti-IgG antibody. The level of ELK1-binded DNA fragments were analysed by Real-time quantitative PCR(RT-qPCR) using the following flanking primers covering Ets-1 element.

The sequences of primers are as below.

ChIP sence 5′ GCCAGGGACTAAAGTGGTGAT 3′;

ChIP antisence 5′ AGTATCCAAGCCCCAGTTGT 3′.

### Luciferase reporter assay

Reporter gene assays were performed in HepG2 cells. The APOA promoter constructs were obtained by PCR amplification. Reporter Plasmid come from YEASEN company (ShangHai, China). HepG2 cells were transfected with full length APOA −2000/+100 promoter reporter plasmid (150 ng). Cells were subsequently treated with DADS for 24 h. Values are normalized to internal control β-galactosidase activity and expressed in percentage.

### Statistical analysis

All data are expressed as mean ± SEM of at least three independent experiments. Results were analysed by ANOVA, followed by Student’s t-test using SPSS13.0 software (IBM Corporation) for comparison. A probability value of *p* < 0.05 was considered statistically significant.

## Results

### DADS downregulate apo(a) expression at the mRNA and protein levels

#### HepG2 cells were cultured in 10% fresh FBS medium for 12 h to achieve synchronised growth

**Fig. 1:**
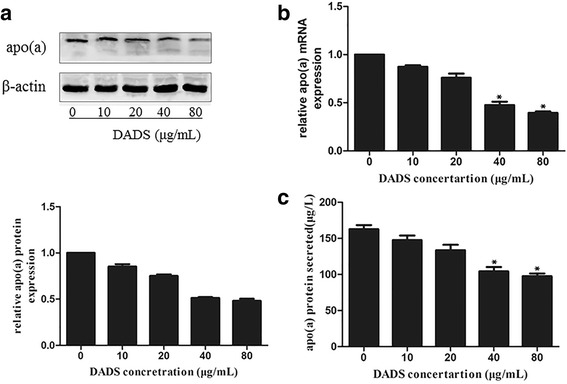
DADS inhibited the expression and secretion of apo(a) in HepG2 cells in a concentration-dependent manner. HepG2 cells were incubated with different doses of DADS (10, 20, 40 and 80 μg/ml) for 24 h. Apo(a) expression in whole-cell lysates from HepG2 cells treated for 24 h with different concentrations of DADS; **a** The expression was analysed using Western blot and normalised to the levels of β-actin. **b** RT-PCR was used to examine the effect of DADS on apo(a) mRNA levels and normalised to β-actin transcripts. **c** Levels of secreted apo(a) in the medium, as determined through ELISA. Each experiment was performed in triplicate, and the results are expressed as mean ± SD, *p < 0.05 versus the control

DADS was dissolved in DMSO and mixed with DMEM for in vitro experiments. The medium was replaced with fresh serum-free medium, and the cells were treated with different concentrations of DADS (10, 20, 40 and 80 μg/mL) for 24 h. DADS remarkably inhibited the mRNA and protein expression of apo(a) and the secretion of apo(a) in a dose-dependent manner. The effects of DADS were observed even at a low concentration of 10 μg/mL, whereas the strongest effect was determined at a concentration of 40 μg/mL (Fig. 1a–c).

#### To determine whether DADS suppressed apo(a) expression and secretion in a time-dependent manner, we incubated HepG2 cells with 40 μg/mL DADS for 0, 6, 12, 24 and 48 h

**Fig. 2:**
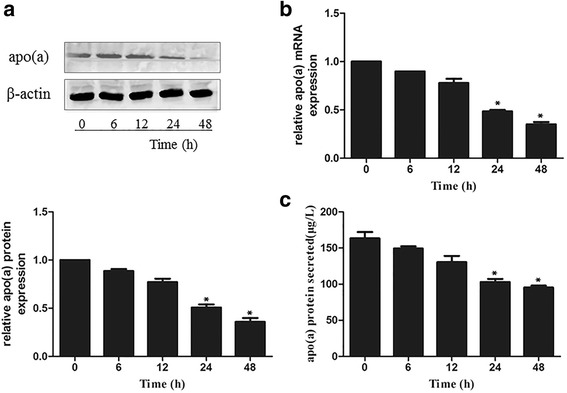
DADS suppressed the expression of apo(a) in HepG2 cells in time-dependent manner. Apo(a) levels in whole-cell lysates from HepG2 cells treated with 40 μg/ml concentrations of DADS for different time (0, 6,12, 24, 48 h). **a** the protein expression was analysed using Western blot and normalised by the levels of β-actin. **b** qRT-PCR was used to examine the effect of DADS on apo(a) mRNA levels and normalised to β-actin transcript levels. **c** Levels of secreted apo(a) in the medium, as determined by ELISA. HepG2 cells were treated with 40 μg/mL DADS for different durations. Each experiment was performed in triplicate, and the results are expressed as mean ± SD, *p < 0.05 versus the control

As shown in Fig. 2a–c, the results of qRT-PCR and Western blot analyses showed that the mRNA and protein levels of apo(a) were significantly downregulated in HepG2 cells after 24 h of incubation with 40 μg/mL DADS (Fig. 2a, b). Expression of apo(a) was reduced, and the strongest effect was observed at 24 h. The secretion levels of apo(a) exhibited the same trends when HepG2 cells were treated with 40 μg/mL DADS for 0, 6, 12 and 24 h (Fig. 2c).

### Phosphorylation of MEK-1 is involved in suppression of apo(a) expression by DADS in HepG2 cells

**Fig. 3:**
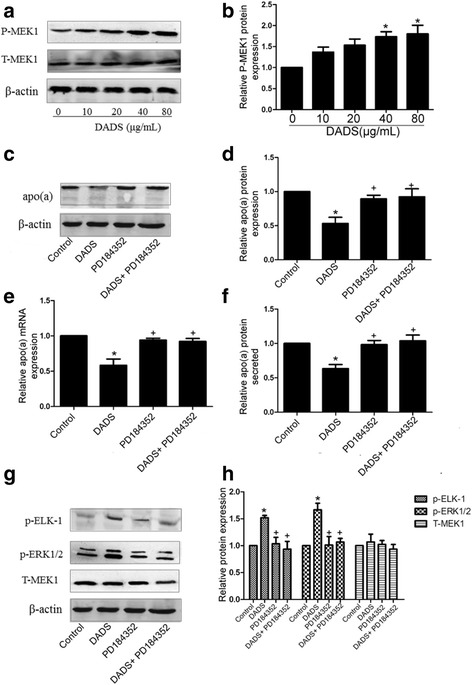
Involvement of MEK1 activation by DADS in apo(a) expression suppression. **a**, **b** DADS increased phosphorylated MEK1 in a dose-dependent manner, as determined through Western blot analysis. HepG2 cells were treated with specific inhibitors of MEK1(PD184352) for 6 h and with DADS (40 μg/mL) for 24 h. **c**, **d** Western blot results and densitometric quantification of apo(a) expression in whole-cell lysates from all groups. **e** The mRNA levels of apo(a) were determined using RT-qPCR. **f** The levels of secreted apo(a) were determined by ELISA. **g**, **h** Western blot results and densitometric quantification of phosphorylated ERK1/2 and phosphorylated ELK1 levels in whole-cell lysates from all groups. Each experiment was performed in triplicate, and the results are expressed as mean ± SD, *p < 0.05 versus control; *p* < 0.05 versus DADS. MEK1 inhibitors were used for PD184352

Western blot analysis was performed to determine whether DADS affects the levels of phosphorylated MEK1 (p-MEK1) in HepG2 cells. The cells were treated with DADS (10, 20, 40 and 80 μg/mL) for 24 h or incubated with DADS (40 μg/mL). As shown in Fig. 3, DADS increased p-MEK1 levels in a dose-dependent manner compared with the control (Fig. 3a, b). We then examined the effect of DADS-mediated inhibition of apo(a) expression through MEK1 signalling. HepG2 cells were treated with specific inhibitors of MEK1(PD184352) to examine whether DADS mediate inhibition of apo(a) expression through MEK1 signalling. The results showed that pre-treatment with PD184352 attenuates the inhibitory effect of DADS on apo(a) expression in HepG2 cells. As shown in Fig. 3, inhibitors of MEK1(PD184352) attenuated DADS-mediated inhibition of apo(a) expression at the mRNA and protein levels (Fig. 3c-f). Meanwhile, inhibitors of MEK1(PD184352) attenuated DADS-induced augment of phosphorylated ERK1/2 and ELK-1(Fig. 3g, h). Thus, DADS downregulated apo(a) expression by regulating MEK-1 signalling.

### DADS suppressed apo(a) expression in HepG2 cells mediated by p-MEK1 protein-stimulated ERK1/2

**Fig. 4:**
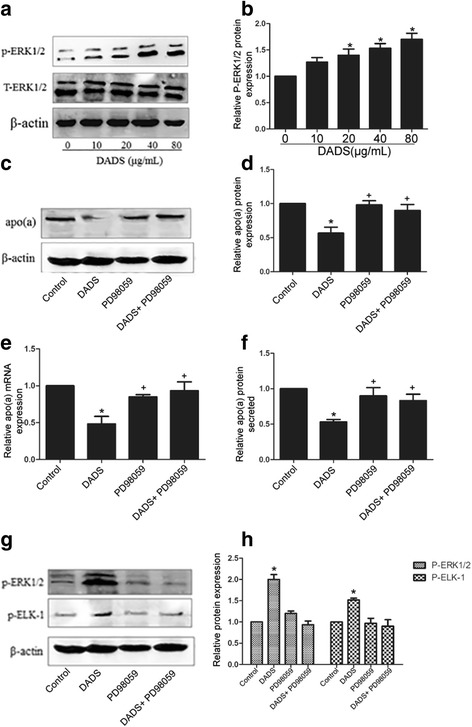
DADS suppressed apo(a) involved in ERK1/2. **a**, **b** DADS activated p-ERK1/2 in a dose-dependent manner, as determined by western blot analysis. **c**, **d**) HepG2 cells were treated with specific ERK1/2 inhibitor (PD98059) for 6 h and with DADS (40 μg/mL) for 24 h. Western blot results and densitometric quantification of apo(a) levels in whole-cell lysates. **e**) mRNA levels of apo(a) were determined by RT-qPCR. **f**) The levels of secreted apo(a) were determined by ELISA. **g**, **h**) Western blot analysis and densitometric quantification of phosphorylated ERK1/2 and phosphorylated ELK1 levels in whole-cell lysates from all groups. Each experiment was performed in triplicate, and the results are expressed as mean ± SD, *p < 0.05 versus control; +*p* < 0.05 versus DADS. ERK1/2 inhibitors were used for PD98059

ERK1/2 is activated by phosphorylated MEK1. To determine whether DADS mediates inhibition of apo(a) expression through p-MEK1-mediated activation of ERK1/2, we evaluated the protein levels of ERK1/2 in HepG2 cells treated with 40 μg/mL DADS for 24 h. As shown in Fig. 4a, western blot analysis indicated that phosphorylation and membrane expression of ERK1/2 were significantly stimulated by DADS (Fig. 4a, b). HepG2 cells were then treated with specific inhibitors of ERK1/2 (PD98059) to examine whether the effect of DADS on inhibition of apo(a) expression is mediated through ERK1/2 signalling. The results showed that pre-treatment with PD98059 attenuated the inhibitory effect of DADS on apo(a) expression in HepG2 cells at the mRNA and protein levels (Fig. 4c-f). Meanwhile, inhibitors of ERK1/2 (PD98059) attenuated DADS-induced augment of phosphorylated ERK1/2 and ELK-1(Fig. 4g, h). These results indicate that DADS downregulate apo(a) expression in HepG2 cells through ERK1/2 signalling.

### DADS suppressed apo(a) expression in HepG2 cells mediated by ELK-1

**Fig. 5:**
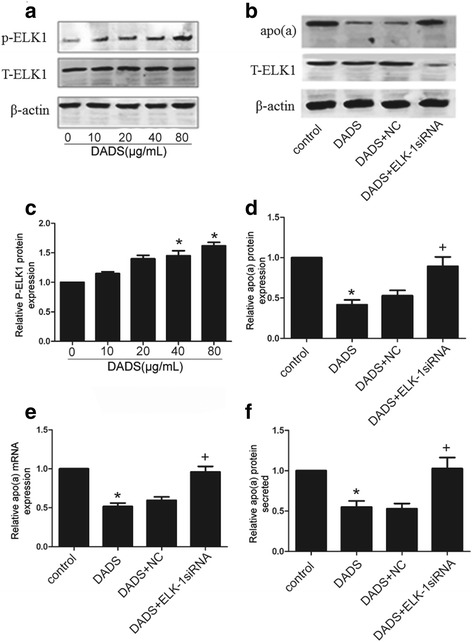
ELK-1 siRNA attenuated the DADS-induced inhibition of apo(a) expression in HepG2 cells. **a**, **b** DADS activated p-ERK1/2 in a dose-dependent manner as determined by western blot analysis. Effects of ELK-1 phosphorylation on inhibition of ELK-1 expression and protein levels of p-ELK-1, as determined by western blot analysis. HepG2 cells were transfected with ELK-1 siRNA or control siRNA (NA) for 12 h before treatment with DADS (40 μg/mL). **c**, **d** Western blot analysis and densitometric quantification of apo(a) levels in protein extracts from each group. **e** mRNA level of apo(a) was determined by qRT-PCR; **f** The levels of secreted apo(a) were determined by ELISA. Each experiment was performed in triplicate, and the results are expressed as mean ± SD. *p < 0.05 versus control, +*p* < 0.05 versus DADS +NC (negative control)

ERK1/2, which can activate ELK-1, is a well-characterised common nuclear substrate that belongs to the family of Ets domain-containing transcription factors. We investigated whether DADS-induced suppression of apo(a) expression in HepG2 cells was mediated by ELK-1. As shown in Fig. 5a, ELK-1 was activated by ERK1/2 phosphorylation (Fig. 5a, c), which was stimulated by DADS. To further characterise the effect of DADS on the downregulation of apo(a) expression, we transfected the cells with ELK-1 siRNA. As shown in Fig. 5b, expression of ELK-1 was suppressed by 92% in HepG2 cells incubated with ELK-1 siRNA for 12 h compared with that in cells treated with mimic negative-control siRNA. Moreover, ELK-1 siRNA partially attenuated DADS-mediated inhibition of apo(a) expression (Fig. 5b–f).

**Fig. 6:**
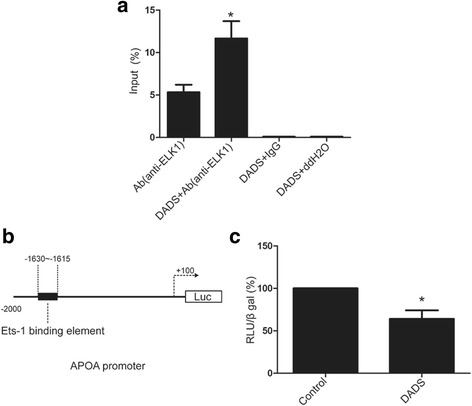
DADS promote ELK1 to bind Ets-1 site of APOA promoter. **a** HepG2 cells were treated with DADS (40 μg/mL) for 24 h. The ELK-1-binded APOA promoter level were analysed by CHIP. Values are normalized to input and expressed in percentage. Each experiment was performed in triplicate. Data are expressed as mean ± SD. *p < 0.05 versus Ab(anti-ELK1) group. **b**, **c** HepG2 cells were transfected with full length APOA −2000/+100 promoter reporter plasmid (150 ng). Cells were subsequently treated with DADS (40 μg/mL) for 24 h. Values are normalized to internal control β-galactosidase activity and expressed in percentage. Data are expressed as mean ± SD. *p < 0.05 versus control group

Ets-1 binding element was identified at −1630/−1615 bp region in the human APOA promoter. This element functions as an ELK-1 binding site that mediates repression of APOA transcription [30]. CHIP and luciferase reporter assay are used to determine whether DADS-activating Elk1 mediate apo(a) expression (Fig. 6a–c).

These data indicate that inhibition of apo(a) expression by DADS is mediated in part via the ERK1/2-ELK-1 pathway in HepG2 cells.

## Discussion

Previous studies have implicated LP(a) as a common and independent risk factor for atherosclerosis. Strong epidemiological evidence showed that LP(a) is an atherogenic lipoprotein. A meta-analysis of 36 prospective studies, involving 126,634 individuals, demonstrated a significant and independent association between plasma LP(a) levels and the risk of cardiovascular morbidity and mortality relative risk for 1 standard deviation (SD) increase in Lp(a): 1.13, confidence interval (CI): 1.09–1.18 [31]. Lamon et al. (2012) showed that CVD risk increased by 2.4-fold after a mean follow-up of 12.3 years in the upper tertile of the baseline plasma Lp(a) levels [32]. Therefore, elucidating the metabolism of LP(a) and apo(a) could facilitate design of effective LP(a)-lowering drugs for patients with high CVD risks. Several pharmacologic agents have been shown to lower Lp(a) including nicotinic acid [33], aspirin [34] and PCSK9 inhibitors [35]. The proprotein convertase subtilisin/kexin type 9 (PCSK9) monoclonal antibody is also known to effectively reduce plasma LP(a) levels [36]. Here, our study showed the ability of DADS decrease apo(a) expression and apo(a) secretion in a time- and dose-dependent manner. Similarly, previous studies demonstrated that DADS is important in decreasing lipid levels, thereby reducing total cholesterol and LDL-cholesterol concentration. Adler et al. (1997) suggested that garlic decreased total cholesterol by 11.5% and LDL-cholesterol by 14.2% [37]. DADS is a natural compound, being enriched in Garlic as a long-term food for humans. Therefore, DADS and garlic has no side effects and is a perfect drug for hyperlipemia.

Plasma LP(a) levels are mainly determined by its rate of biosynthesis. Wade et al. (1993) demonstrated that the apo(a) promoter region, which extends from −98 bp to +130 bp, functions both as a negative enhancer region and a positive enhancer, and contains an HNF1A-binding site related to the mRNA start site [38]. Negi et al. (2004) reported the locations of a negative enhancer (−1432 bp to −716 bp) region and a tissue-specific (−716 bp to −616 bp) region, that regulate apo(a) gene expression. Additionally, the transcription factors GATA4 and HNF3A were also discovered to bind and repress apo(a) expression [39]. Chennamsetty et al. (2011) reported that the Farnesoid X receptor (FXR) can bind to a negative control element DR-1 located in the −826 bp to −814 bp region of the apo(a) promoter [40]. Thus, FXR activation can reduce plasma concentrations of LP(a) and liver expression of human LPA. Furthermore, Chennamsetty et al. (2012) identified a signalling pathway involved in the nuclear translocation of ELK-1, which binds to the promoter region of apo(a) at −1630 bp to −1615 bp and represses apo(a) transcription [40]. Here, our study also showed ELK-1 can bind to the promoter region of apo(a) at −1630 bp to −1615 bp and inhibit apo(a) expression.

There are so many published papers showing directly or indirectly that DADS interfere in HepG2 cells with the MAPK pathway. Ji et al.(2010a) indicate that p38MAPK and caspase-3 are involved in the process of DADS-induced apoptosis in human HepG2 cells and interact with each other [37]. Luo et al. (2015) suggested that DADS can activate phosphorylation of the ERK1/2 signalling pathway in a dose-dependent manner in HepG2 cells [41]. Moreover, ELK-1, a member of the family of Ets domain-containing transcription factors, is a well-characterised common nuclear substrate for activating ERK1/2. Phosphorylated ELK-1, which is transported from outside the nucleus to the nucleus, can bind to the Ets-1 motif of the apo(a) promoter to repress apo(a) expression. Here, our results showed DADS can activate phosphorylation of MEK1/ ERK1/2 and further activate the phosphorylation of ELK-1. Then Phosphorylated ELK-1 is transported from outside the nucleus to the nucleus and bind to the Ets-1 motif of the apo(a) promoter to repress apo(a) expression. We also use a specific MEK inhibitor, an ERK1/2 inhibitor (PD98059), and ELK-1 siRNA to further determine the role of MEK1, ERK1/2 and ELK-1on apo(a) expression. As shown in Figs 3, 4, and 5, MEK1, ERK1/2 and ELK-1are necessary to DADS-mediated inhibition of apo(a) expression. Thus, the effect of DADS on HepG2 cells was found to be mediated in part through the MEK-ERK1/2-ELK-1 pathway.

## Conclusion

We found that DADS repress apo(a) expression in HepG2 cells, and the effect is mediated partially through the MEK-ERK12-ELK-1 pathway. Thus, our findings provide a new direction for the development of LP(a)-lowering agents. Further studies must be performed to identify whether DADS inhibit apo(a) expression in vivo and to elucidate the interaction among transcription factors involved in the process.

## Abbreviation

Apo(a): Apolipoprotein (a)
ApoB: Apolipoprotein B
AS: Atherosclerosis
CVD: Cardiovascular disease
DADS: Diallyl disulphide
DMEM: Dulbecco’s modified Eagle’s medium
ELISA: Enzyme-linked immunosorbent assay
ERK: Extracellular regulated protein kinases
LDL: Low-density lipoprotein
LP(a): Lipoprotein(a)
MAPK: Mitogen-activated protein kinases
PCSK9: Proprotein convertase subtilisin kexin type 9.
siRNA: Small interfering RNA.
TC: Total cholesterol.
